# Application Research of Tooth Arrangement Based on Rotation Matrix Calculation and Resistance Detection in Oral

**DOI:** 10.1155/2022/4675181

**Published:** 2022-05-20

**Authors:** Mingming Wu

**Affiliations:** Engineering Laboratory for Biomaterials and Tissue Regeneration, Ningbo Stomatology Hospital, Ningbo 315000, China

## Abstract

The goal of this research was to provide a new approach for analyzing orthodontic teeth arrangement inside oral depending on the rotation matrix computation and resistance detection. The present method includes the following operations within a certain therapy period: first three-dimensional positions of the tooth were evaluated with a pierced laser beam and a three-dimensional system of surface-scanning. Second, the three-dimensional shape data was automatically registered at maxillary 1^st^ molars, and methods of coordinate had been normalized. Third, a translation vector and rotation matrix had been evaluated from automatic registration of two position data of a particular tooth. Fourth, the limited spiral axes of teeth had been measured as the zero rotational dislocation locus; and impressions for a model of the dental cast had been taken at five different points: shortly before and after device was fitted, and ten days, one month, and two months after the treatment started. The results showed that existing analysis approach could more quickly classify a specific tooth's movement by spinning all over and translating along a finite helical axis. It can provide statistical visual three-dimensional data on complex tooth arrangement throughout orthodontic therapy.

## 1. Introduction

The orthodontic tooth arrangement is a complicated biological process characterized by the periodontal tissue's gradual reactions towards the biomechanical stimuli [1, 2]. The size, direction, and moment–force ratio of the force applied and physiological state of each patients' periodontal tissue substantially influence tooth arrangement [3–5]. Even though such studies only used a one or 2-D investigation, other morphometrical and theoretical studies about tooth arrangement have provided considerable information [6–9]. Moreover, all these investigations only explain the ﬁrst tooth arrangement even before periodontium undergoes degenerative modification [10–13]. A statistical analysis utilizing the finite-element method produces a three-dimensional stress distribution throughout periodontal tissue and three-dimensional tooth dislocation under different loading conditions [13, 14].

**Figure 1:**
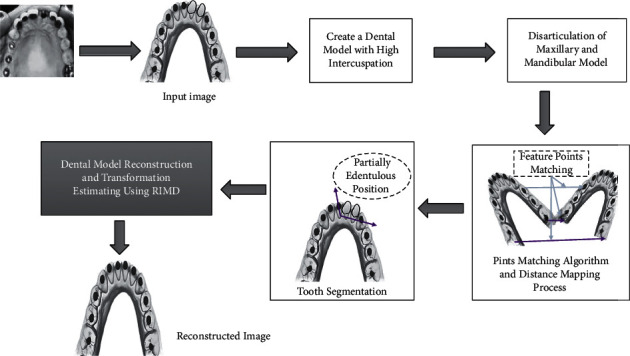
Overall design of reconstructed-based identical matrix point.

The aim of this research had to create a new approach to evaluate the orthodontic teeth arrangement focusing on the calculation of rotation matrix, focusing on the precision of three-dimensional surface measurement and analysis of limited helical axis, for providing health-care professionals with accurate visual information [15, 16]. The main features of orthodontic treatment include jaw relation, tooth size, and tooth alignment [17, 18]. An oral cavity is examined, radiographs are evaluated, and dental casts are evaluated in order to collect the essential data for treatment decisions [19, 20]. However, from the perspective, there are limitations in correctly seeing the palate, lingual surface, and occlusal [21]. Furthermore, imprint treatments may be painful for the patient and need more chair duration [22, 23]. In addition to the logistical and financial challenges, the plaster casts require physical storage space. Furthermore, dental plaster casts visual examination does not allow physicians to check, measure, or monitor orthodontic tooth arrangement and the root region of surrounding bone [24, 25]. With each subsequent activation of the device, the application of orthodontic force via the appliance adjusts the teeth in progressive stages [26]. As shown in (Figure 1), symmetrical characteristics of the dentition are used in the reconstructed-based identical matrix point approach, a novel method of analyzing tooth arrangement [27].

## 2. Literature Review

Saratti et al. [28] conducted a comprehensive literature search employing PubMed, a database of Cochrane Library, and Google Scholar. They provided up-to-date details on two issues. The first one was how natural tooth tissues combined to make architecture as strong, tough, and resistant to strain faults the tooth. The second was how ‘bio-inspiration' was being implemented to develop and manufacture restorative dentistry while considering the limitations of existing dental methods. They expressed that bio-inspired principles had previously been effectively employed to improve the strength and toughness of artificially made materials in various engineering sectors. They highlighted that three-dimensional printing techniques also provide a novel and promising avenue for rebuilding dental tissues. Anil et al. [27] used a reconstructed-based identical matrix point (RIMP) approach to build a new way for re-establishing dental occlusion. They rebuilt the curvature of dental areas utilizing distance mapping to save calculation time. They also employed a technique of iterative point matching for precise re-establishment. They used a setup of dental experimental with high-quality digital camera pictures to examine satisfactory restoration and occlusion testing. Their suggested RIMP outperformed traditional approaches like GLCM, Fuzzy C Means, PCR, OGS, and OPOS in terms of the overall accuracy of 91.50 percent and an efficiency of 87.50 percent.

Schneider et al. [29] proposed optical coherence tomography, a revolutionary image-based approach that had significant potential in aiding regular tooth examination. They expressed that the cross-sectional pictures acquired were simple to comprehend and process. They observed that multiple uses of OCT in cariology had been studied, ranging from detecting various problems to restoration monitoring and reporting or the visualization of therapy processes. Their review based on chosen cases described the potential and limits of their approach in cariology and restorative dentistry, which were the most clinically essential domains of dentistry. Patil et al. [21] focused on polylactic acid, acrylonitrile styrene acrylate, polymethylmethacrylate, and limpet teeth (a mixture of chitin and goethite). They investigated the performance of novel materials using analytical and experimental approaches. However, they expressed that J-OCTA software had overcome experimentation-related difficulties such as costs and time. They concluded that compared to other simulation tools, this approach worked on the molecular dynamics principles to study the efficiency of the soft materials having more precision. They highlighted that the experimental techniques provide erroneous findings, while the analytical methods are confined to smaller materials because each particle has rotational and translational velocities.

Cho et al. [25] used atomistic simulations and sole fiber/microdroplet pull-out experiments in micro-scale to undertake a comparative study on the behavior of interfacial adhesion of short glass fiber and matrix of dental resin. They determined the interfacial shear strength at the molecule level by adding a factor of scale to the glass fiber. They compared the simulation findings to experimental findings of pull-out experiments. They expressed that both results confirmed the improved, reinforcing effects of modifying the surface with agents grafting of silane coupling on glass fibers. Furthermore, they investigated the mechanical characteristics and dynamic behavior of dental materials under transverse and longitudinal tension loadings that were using free volume variations. The results of their study provided optimum design recommendations for accurately predicting the mechanical properties of short fiber-reinforced dental composites using simulations, molecular dynamics, and testing. Lopez et al. [30] used a laboratory test that replicated the clinically apparent wear aspects to investigate wear processes inside a dental materials suite with a ceramic element and tooth enamel. They employed a tetrahedral ball-on-3-specimen analyzer with a revolving challenging opponent zirconia sphere to induce circular wear marks on the surface of dental composites using artificial saliva. They expressed that wear scars images allowed for the analysis of wear processes, while measurements of scar dimension quantify abrasive wear. They explained that Zirconia ceramics had the lowest rates, while lithium disilicate had the highest, having feldspathic ceramics and ceramic–polymer composites in the middle. They discussed that examination of fatigue scars showed surface residues, indicating a material removal process at the microstructural level. They accounted for mild and severe wear zones for utilizing microcracking and microplasticity models. They also used *w* ear models to assess the possible lifespan of various dental materials. They concluded that wear damage would produce significant material loss, resulting in early tooth or prosthesis failure.

Tahir et al. [31] suggested and tested a simulator of mechanical mastication that would simulate the force cycle of human rumination and record the requisite interactive loading via specially constructed force sensors. Their suggested method made the tooth-replacement surgery easier. They discussed that PKM completed a mastication cycle with six degrees of freedom, allowing any movement and rotation in the horizontal, vertical, and sagittal planes. Their proposed mechanism had a force transmission range of approximately 2000 N and would imitate the mastication cycle of humans. Their constructed load-sensing device would capture interactive forces ranging from 200 N to 2000 N using rapid reaction and high sensitivity to establish a simulator mechanical mastication using custom-made modules. Wei et al. [26] presented a learning-based strategy for quickly and automatically arranging teeth. They constructed the task of tooth arrangement as a unique structural 6-DOF pose estimation problem and resolved it by presenting a novel neural network structure to train from a vast number of clinical studies encoding successful orthodontic treatment instances. They claimed that extensive studies had confirmed their strategy, which yields promising descriptive and analytical results.

Zhang et al. [32] proposed a multi-manipulator tooth arrangement approach for full denture production. They suggested a revolutionary entire denture production method using a generator of dental arch and a multi-manipulator. They used an analytical technique to build the kinematics version of tooth arrangement of a multi-manipulator robot based on the concept of tooth arrangement for a complete denture. Their proposed multi-manipulator tooth-arrangement robot was used for preliminary tooth-arrangement tests. As per the jaw arch specifications, their multi-manipulator tooth-arrangement robot can autonomously design and produce a set of complete dentures for a patient. Their experimental findings confirmed the validity of the kinematics concept of the multi-manipulator tooth-arrangement robot and the viability of the whole denture manufacturing strategy implemented by the multi-manipulator tooth-arrangement robot. Cheng et al. [33] explained a newly created virtual, customized, and precise tooth arrangement mechanism based on comprehensive dental root and skull information. They made a feature-limited database of a three-dimensional tooth model. Second, they established anatomical points of reference, the reference lines and planes for tooth movement computational simulation. They thoroughly exploited the corresponding mathematical formalism of the tooth pattern and the concept of the stiff body's unique posture transformation. Their experimental findings suggested that the approach of virtual tooth arrangement would successfully arrange aberrant teeth and was suitably flexible. Their newly designed method was distinguished by its high-speed processing and quantitative measurement of each tooth's level of three-dimensional movement.

## 3. Methods

**Figure 2:**
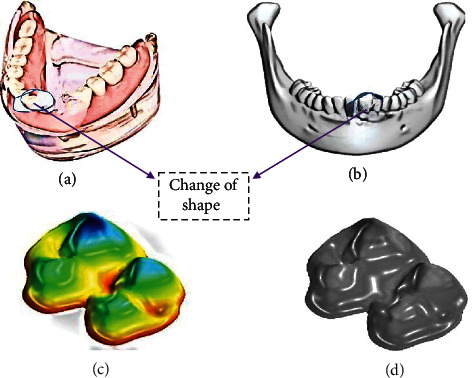
(a) Full mandibular model. (b) A component of mandibular model chosen. (c) The triangular mesh's foundation. (d) Cloud point as seen with the MATLAB-based toolbox.

To put the existing procedure to the test, a male patient of 22 yr two months had been examined in the Dental Hospital. The hospital treated the patient's Angle Class III malocclusion with anterior teeth modest crowding with a tool of multi-bracket. Conventional hooks with edge of 0.460.64 mm standard slot size were used for the primary leveling and arrangement, together with 0.41 mm hardened Ni–Ti round wire as well as a rectangular wire of 0.41 mm to 0.410.56 mm. The paired maxillary initial molars related to a bar of transpalatal to improve anchoring as well as provide immovable reference features for superimposition. The models of *d* dental cast were generated at each step employing alginate impressions made by die stone. A model of complete mandibular is presented in Figure 2.

### 3.1. Printing Models of Dental Cast

**Figure 3:**
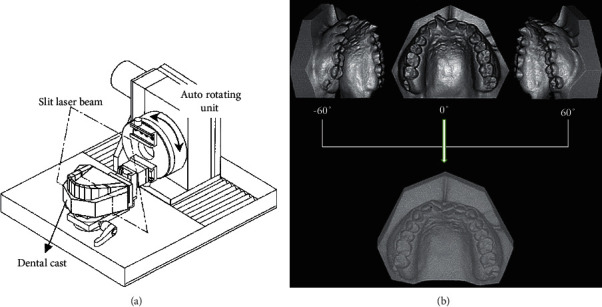
(a) Three-dimensional strategy of surface scanning and a three-dimensional scanned shape of maxillary dental casts (b).

Prints for models of the dental cast took 5 times: instantly before and after the appliance has been implanted (T0 and T1), ten days (T2), one month (T3), and two months with (T4) right after treatment started. To test the dental cast reproducibility, a model of proxy dental with the marks of hemisphere (reentrants; 3 mm in diameter) to the maximal incisor of right central and bilateral maximal first molars has been used as a template. A point of reference was placed inside the center point of every re-entrant. We created five alginate impressions, as well as die stone cast replicas. The points of reference distance were calculated 5 times on prototype as well as five duplicate models using a three-dimensional CNC of high-precision measurement machine. The average distance disparities between the duplicate models and prototype were calculated. The dental castings were calculated with a three-dimensional surface-scan machine equipped with the splitting laser beam known as VMS-150RD, UNISN, Japan. The system included a slicing laser projector, 2 CCD (charge-coupled device) cameras, a mounting unit with the auto-rotating feature, and one PC (Figure 3(a)). An *X*-axis resolution had been 0.01 mm, while the 0.1 mm resolution of the *Y*-axis.

A three-dimensional shape system of data-analysis composed of (Zx1, Intergraph) graphic workstations (Surfacer, Image ware) data-processing and -analyzing software and (Visual *C*++ 5.0, Microsoft), a newly constructed numerical and analytical program was utilized to demonstrate the limited helix axis.

**Figure 4:**
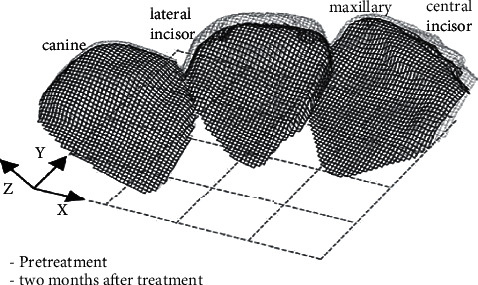
Before and after treatment view of a normalized three-dimensional anterior teeth. Black represents before treatment, and gray represents two months after the therapy.

Dental casts were scanned with three orientations to decrease blind areas by rotating the auto-rotational positioning unit of the computational tools. The post-processor combined the three data files into a single file (Figure 3(b)). It was used to test the measurement precision of the three-dimensional measuring equipment by measuring a calibration plane plate, and the best-fitted equation for plane was derived on all data inside about 73 seconds utilizing the least-squares approach. The most significant divergence between the projected plane and the actual data was used to assess the measurement accuracy. The placement of dental cast on auto-rotating modules remained random while scanning. A standard coordinate system must be built to predict tooth-arrangement three-dimensionally relying on the three-dimensional shape. Many fixed components known to every model had been chosen as superimposition points of reference. This study selected the maxillary 1st molars as registered components as they had been relatively stable after treatments. Registration conducted automatically (automatically, registration was built-in Surfacer software function).  Figure 4 depicts the pre- and post-treatment normalized three-dimensional shape data for maxillary teeth.

### 3.2. Difference between Maxillary and Frontal Teeth Normalized Images

**Figure 5:**
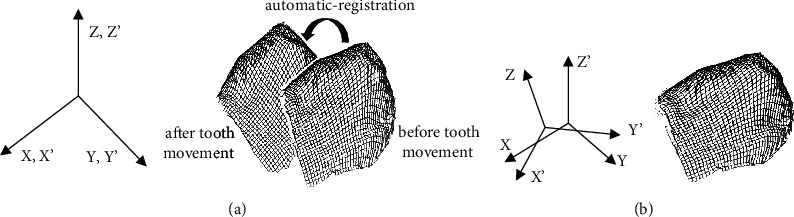
A coordinate system of automatic registration for assessing tooth arrangement is known as before-&-after registration. The coordinate systems (*x*0, *y*0, *z*0) and *P*(*x*,*y*,*z*) adhere to tooth arrangement before-&-after.

The difference between maxillary and frontal teeth normalized photos was defined as the tooth arrangement. A vector of translation and a matrix of rotation generated by adding the three-dimensional shapes before as well as after tooth arrangement could be utilized in (Figure 5) to represent the arrangement of a single tooth. Employing the least-squares technique to investigate the error of mean fitting for an automated registration of three-dimensional structures, a mean three-dimensional distance with every tooth point towards its closest neighboring point seen between points' sequences on the two registered forms was determined for every treatment point [34]. In the existing investigation, we employed a minimization strategy utilizing commercially using software to build a rotation matrix from autonomously recorded location data, and after, we also used the resulting matrix to predict the helical axis. After movement, every point of *P* (*x*^0^, *y*^0^, *z*^0^) on the three-dimensional form was represented in the system of coordinate *X*, *Y*, and *Z* as a factor of six translations as well as rotation constants. So, Figure 3 depicts the three-dimensional dislocation of *P*(*x*,*y*,*z*) with three-dimensional form prior to movement.

The formula is determined by

(1) $$\begin{array}{c} m=An+o, \end{array}$$

where A depicts the 33-rotation matrix, *m* represents the arbitrary point position vector after the tooth arrangement, *n* shows the position vector of arbitrary point before arrangement, and *o* depicts translation vector. After that, tooth arrangement in three-dimensional space was identified as a *p* displacement vector with (*x*′ − *x*),  (*y*′ − *y*),  & (*z*′ − *z*) components. This motion includes both rotation and translation. Equation (2) is used to describe the (P) displacement vector.

(2) $$\begin{array}{c} p=\left(A-K\right)n+o. \end{array}$$

*K* depicts the unit matrix that is also an initial coordinate's function. The 33 matrix A represents the all-around rotation axis that passes through the origin as well as parallel to a limited helical axis. Because the displaced vector *p* only runs parallel towards the limited helical axis and therefore is undisturbed by rotations for locations on this axis, this axis position has been given by

(3) $$\begin{array}{c} p=A^{T}p. \end{array}$$

As a result, Equation (2) is multiplied by the AT and also applying the relationship within Equation (3).

(4) $$\begin{array}{c} \left(A-K\right)n+o=\left(A-K\right)n+A^{T}o. \end{array}$$

Equation (4) becomes

(5) $$\begin{array}{c} \left(A+A^{T}-2K\right)n+\left(K-A^{T}\right)o=0. \end{array}$$

Equation (5) becomes

(6) $$\begin{array}{c} \frac{d}{db}=\left\{\left(\left(A-K\right)n+o^{T}\left(A-K\right)n+o\right)\right\}=0. \end{array}$$

This produces the locations *n* with the smallest displacements and resides on the limited helical axis. The relationship in Equation (6) holds at all positions along the helix axis. Though not distinct, the solution may be achieved by swapping the value for the component of *Z*.

When the *b* has been selected on a limited helix axis, as calculated by Equation (1), it is also on the same axis. A translation *p* with a limited helical axis equals to the distance between *a* and *b*. Following that, an arbitrary point*d*_0_ was picked outside of a helix axis, and also its location after arrangement of tooth *d*_1_ had been determined once again using Equation (1):

(7) $$\begin{array}{c} d_{1}=Ad_{0}+o. \end{array}$$

Further, the *d*_0_ and *d*_2_ (*d*_2_=*d*_1_ − *v*) orthogonal projection on helical axis calculated as

(8) $$\begin{array}{c} q^{\prime }=q+\frac{\left(d-q\right).h}{h.h}h, \end{array}$$

wherein q^0^ is a point's position vector on the helical axis, *q* depicts any point's position vector on helix axis, *d* is either *d*_0_ or the *d*_2_, and the helical axis direction. Thus, this angle of rotation within space around a limited helical axis is just like angle among *d*_0_, q^0^, and *d*_2_.

## 4. Results

**Figure 6:**
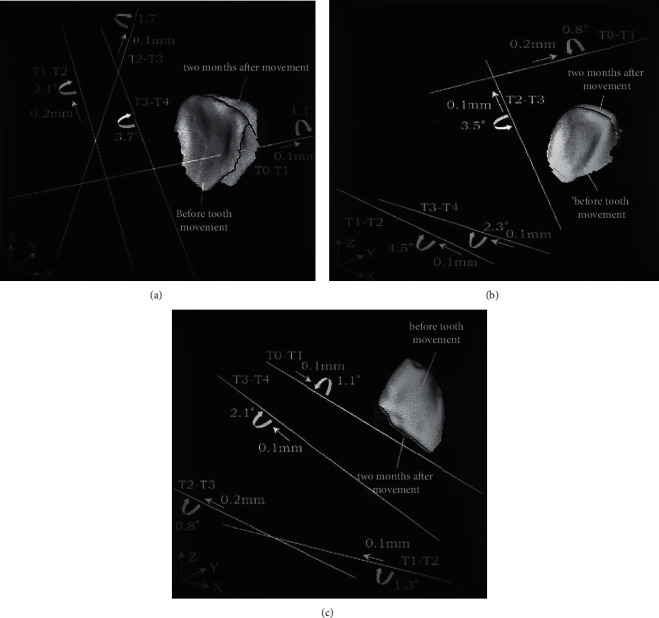
Graph depicting the three-dimensional displacement of the maxillary right centralized incisor, canine as well as lateral incisor during two months of therapy. The angle of rotation and amount of translation with a helical axis is++ used to indicate tooth movement. T0: shortly before device application; T1: instantly after device implementation; T2: Ten days of treatment; T3: one month of treatment; T4: Two months after therapy. The white bar represents the rotation axis; the curved arrow represents the rotation angle; and the little straight arrow represents the translation amount.

Dental cast means the error was 0.07 mm from the incisor of right-center towards right 1st molar, 0.05 mm towards left 1st molar, and 0.04 mm for both the right and left 1st molars, showing that the die stone had slightly expanded during the setting process. Their standard deviations remained 0.04 mm or less. The three‐dimensional measuring instrument has a seven 0.05-mm measurement accuracy. The lateral incisor, centralized incisor, and canine had to mean fitting errors of 0.05, 0.05, and 0.04 mm, correspondingly (Table 1). The helix axis of the right central incisor would be within the crown and almost orthogonal to lingual surface of crown throughout T0 to T1 (Figure 6(a)). An angle of helical axis rotation had been 1.11, and the lingual translation became 0.1 mm. In the first ten days of therapy, this helical axis migrated almost orthogonal to the occlusal plane (T1 to T2). Their occlusal translation approximated 0.2 millimeters and 2.11 degrees of the rotation angle. From ten days to one month after therapy (T2 to T3), the helix axis migrated labially and was almost parallel to tooth's long axis. The occlusal displacement gave 0.1 millimeters and 1.71° rotation angle. Their helical axis migrated palatially from one to two months after therapy (T3 to T4) and was approximately parallel to a helical axis through T1 to T2.

The translation was minimal, and the angle of rotation was 3.71. These data imply that immediately after the device was implanted, the center incisor translated considerably labially along with palatal tilt, then twisted histologically and pointed labially with the intrusion. The maxillary helical axis of the right lateral incisor was outside from crown and also was about 451 to the tooth's long axis from T0 to T1 (Figure 6(b)). The labio-occlusal translation measured value was 0.2 millimeters, and the rotation angle became 0.81°. From T1 to T2, a lateral incisor migrated significantly. At the root surface, the helix axis changed and nearly paralleled to the occlusal plane. Their distal translation approximated 0.1 millimeters, and the rotation angle was 4.51°. The helix axis of the tooth migrated towards the disto-palatal axis with a twist of 3.51 and an occlusal movement of 0.1 millimeters from T2–T3. T3–T4 saw a similar shift in the helical axis of T1–T2. The distal translation approximated 0.1 millimeters, and the angle of rotation became 2.31°. Its lateral incisor first inclined palatal, later labially by the intrusion, twisted mesio-labially, and ultimately slanted labially with invasion with these data. The helical axis of maxillary right canine was nearly parallel towards an occlusal plane during T0–T4, while direction of rotation and translation altered after T1 (Figure 6(c)). These actions included jiggling of labio-lingual.

## 5. Discussion

In this work, we used a screw axis, also known as a helical axis, to measure the three-dimensional motion of a solid body to investigate the arrangement of specific teeth compared to standard teeth [35, 36]. Woltring cross-validation method [37] illustrated that the angle of rotation and translation has become relatively well-focused. Still, the direction and position of the helix axis has been greatly sensitive to calculations errors of landmarks, particularly for the little rotations, the long distances towards the center of gravity mean of the monuments, and also small monument sizes of distribution. In the current work, we studied dental cast repeatability, the measurement precision of three-dimensional system of surface scanning, and a maximum superimposition error employing least-squares technique, and all errors would be within 0.05 millimeter range.

**Figure 7:**
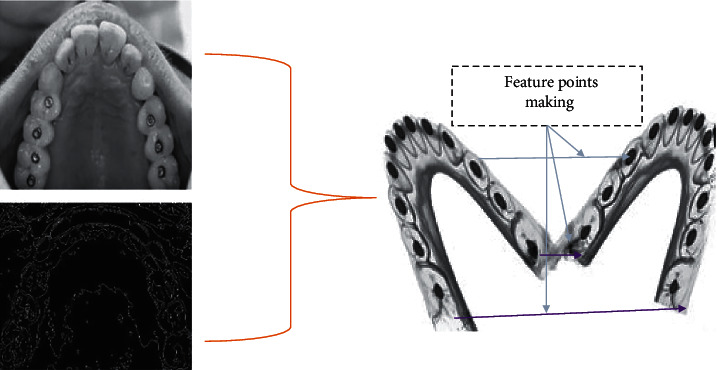
Proper articulation of dental prototypes.

The number of orthodontic tooth arrangements differs across patients and therefore is determined by the force direction, moment–force ratio, force magnitude, and periodontal tissue quality [31]. The horizontal arrangement of a buccal cusp points of the maxillary premolars under a constant force of 0.5 N was calculated towards being 1.7 mm (ranging 0.5–3.4 mm) and 4.3 mm (range 2.7–7.1 mm), correspondingly, after 4 and 7 weeks. After four weeks, the tooth arrangement varied from 0.2 to 2.2 mm when a pressure of 1 to 1.5 N had been implemented towards the maxillary canine [38]. As a result, the current method's accuracy is enough to examine the orthodontic tooth arrangement. As shown in (Figure 7), when the feature points of camera photos are matched, the dental prototypes are appropriately articulated.

**Figure 8:**
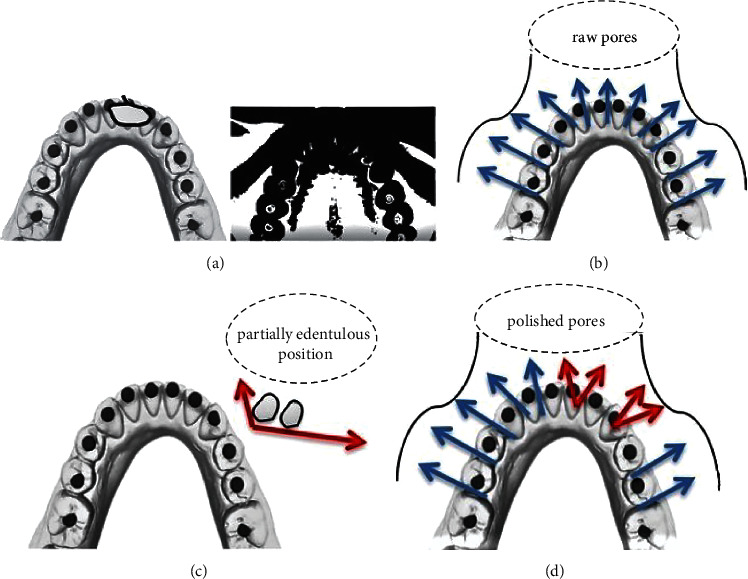
(a) Image selection for dental image classification. (b) Unprocessed image. (c) Edentulous area. (d) Extensive sequence.

Translation (movement of the body), rotation (tipping motion), or a mixture of the two is caused by orthodontic force. The model of tooth arrangement is established by how the force's route of action correlates to the teeth's center of reluctance [27]. Traditionally, 1 or 2 two locations in the teeth were selected as points of reference [39], and the center of rotation position defined the final tooth arrangement [40]. When a horizontal force is given to the lingual surface, it induces simple rotation (tipping) along the root's bottom half. The present research clearly shows that the arrangement of orthodontic tooth is complex and varies greatly from past study data, specifically in severe crowding. The original tooth position and force applied to the teeth, and the contact conditions and movement behavior of surrounding teeth all influence orthodontic tooth arrangement. Surprisingly, the rotational axis position changed significantly throughout treatment stages, even though this was assumed that the rotational axis location would change gradually and consistently during tooth arrangement. Nevertheless, part of the axis variability might be attributable to measurement mistakes. A complete investigation of the accuracy of the axis parameters utilizing a new setup of the experiment would be required. The four stages of tooth arrangement and sequences are shown in Figure 8.

In this work, we created a new way to analyze orthodontic tooth arrangement that may provide orthodontists with accurate visual data on the tooth arrangement and may be a helpful strategy for developing an orthodontic technique. Moreover, three-dimensional data on teeth arrangement during therapy would enable orthodontic tooth-arrangement modeling, which might be included in treatment planning. The better architecture and the cheap treatment of orthodontic tooth arrangement would be an area of interest in future.

## Figures and Tables

**Table 1:** Maximum errors also known as fitting error for the automated registration employing the method of least squares (mm).

Stages	Canine	Central incisor	Lateral incisor
T0–T1	0.05	0.05	0.05
T1–T2	0.04	0.04	0.04
T2–T3	0.03	0.02	0.02
T3–T4	0.02	0.03	0.03
Mean	0.04	0.05	0.05

## Data Availability

The labeled datasets used to support the findings of this study are available from the corresponding author upon request.
